# The origin of life: what we know, what we can know and what we will never know

**DOI:** 10.1098/rsob.120190

**Published:** 2013-03

**Authors:** Addy Pross, Robert Pascal

**Affiliations:** 1 Department of Chemistry, Ben-Gurion University of the Negev, Be'er Sheva, 84105, Israel; 2 Institut des Biomolécules Max Mousseron (UMR 5247, CNRS, Universités Montpellier 1 and Montpellier 2), Université Montpellier 2, Place E. Bataillon 34095, Montpellier Cedex 05, France

**Keywords:** abiogenesis, origin of life, dynamic kinetic stability, systems chemistry

## Abstract

The origin of life (OOL) problem remains one of the more challenging scientific questions of all time. In this essay, we propose that following recent experimental and theoretical advances in systems chemistry, the underlying principle governing the emergence of life on the Earth can in its broadest sense be specified, and may be stated as follows: all stable (persistent) replicating systems will tend to evolve over time towards systems of greater stability. The stability kind referred to, however, is dynamic kinetic stability, and quite distinct from the traditional thermodynamic stability which conventionally dominates physical and chemical thinking. Significantly, that stability kind is generally found to be enhanced by increasing complexification, since added features in the replicating system that improve replication efficiency will be reproduced, thereby offering an explanation for the emergence of life's extraordinary complexity. On the basis of that simple principle, a fundamental reassessment of the underlying chemistry–biology relationship is possible, one with broad ramifications. In the context of the OOL question, this novel perspective can assist in clarifying central ahistoric aspects of abiogenesis, as opposed to the many historic aspects that have probably been forever lost in the mists of time.

## 2. Introduction

The origin of life (OOL) problem continues to be one of the most intriguing and challenging questions in science (for recent reviews on the OOL, see [1–6]). Its resolution would not only satisfy man's curiosity regarding this central existential issue, but would also shed light on a directly related topic—the precise nature of the physico-chemical relationship linking animate and inanimate matter. As one of us (A.P.) has noted previously [1,7,8], until the principles governing the process by which life on the Earth emerged can be uncovered, an understanding of life's essence, the basis for its striking characteristics, and outlining a feasible strategy for the synthesis of what could be classified as a simple life form will probably remain out of reach. In this essay, we will argue that recent developments in systems chemistry [9–11] have dramatically changed our ability to deal with the OOL problem by enabling the chemistry–biology connection to be clarified, at least in broad outline. The realization that abiogenesis—the chemical process by which simplest life emerged from inanimate beginnings—and biological evolution may actually be one single continuous physico-chemical process with an identifiable driving force opens up new avenues towards resolution of the OOL problem [1,7,12,13]. In fact that unification actually enables the basic elements of abiogenesis to be outlined, in much the same way that Darwin's biological theory outlined the basic mechanism for biological evolution. The goal of this commentary therefore is to discuss what aspects of the OOL problem can now be considered as resolved, what aspects require further study and what aspects may, in all probability, never be known.

## 3. Is the origin of life problem soluble in principle?

In addressing the OOL question, it first needs to be emphasized that the question has two distinct facets—historic and ahistoric, and the ability to uncover each of these two facets is quite different. Uncovering the historic facet is the more problematic one. Uncovering that facet would require specifying the original chemical system from which the process of abiogenesis began, together with the chemical pathway from that initiating system right through the extensive array of intermediate structures leading to simplest life. Regretfully, however, much of that historic information will probably never be known. Evolutionary processes are contingent, suggesting that any number of feasible pathways could have led from inanimate matter to earliest life, provided, of course, that those pathways were consistent with the underlying laws of physics and chemistry. The difficulty arises because historic events, once they have taken place, can only be revealed if their occurrence was recorded in some manner. Indeed, it is this historic facet of abiogenesis that makes the OOL problem so much more intractable than the parallel question of biological evolution. Biological evolution also has its historic and ahistoric facets. But whereas for biological evolution the historic record *is* to a degree accessible through palaeobiologic and phylogenetic studies, for the process of abiogenesis those methodologies have proved uninformative; there is no known geological record pertaining to prebiotic systems, and phylogenetic studies become less informative the further back one goes in attempting to trace out ancestral lineages. Phylogenetic studies presume the existence of organismal individuality and the genealogical (vertical) transfer of genetic information. However, the possibility that earliest life may have been communal [14] and dominated by horizontal gene transfer [15–17] suggests that information regarding the evolutionary stages that preceded the last universal common ancestor [18] would have to be considered highly speculative. Accordingly, the significance of such studies to the characterization of early life, let alone prebiotic systems, becomes highly uncertain.

The conclusion seems clear: speculation regarding the precise historic path from animate to inanimate—the identity of specific materials that were available at particular physical locations on the prebiotic Earth, together with the chemical structures of possible intermediate stages along the long road to life—may lead to propositions that are, though thought-provoking and of undeniable interest, effectively unfalsifiable, and therefore of limited scientific value.

Given that awkward reality, the focus of OOL research needs to remain on the *ahistoric* aspects—the principles that would explain the remarkable transformation of inanimate matter to simple life. There is good reason to think that the emergence of life on the Earth did not just involve a long string of random chemical events that fortuitously led to a simple living system. If life had emerged in such an arbitrary way, then the mechanistic question of abiogenesis would be fundamentally without explanation—a stupendously improbable chemical outcome whose likelihood of repetition would be virtually zero. However, the general view, now strongly supported by recent studies in systems chemistry, is that the process of abiogenesis was governed by underlying physico-chemical principles, and the central goal of OOL studies should therefore be to delineate those principles. Significantly, even if the underlying principles governing the transformation of inanimate to animate *were* to be revealed, that would still not mean that the precise historic path could be specified. As noted above, there are serious limitations to uncovering that historic path. The point however is that if the principles underlying life's emergence on the Earth could be more clearly delineated, then the mystery of abiogenesis would be dramatically transformed. No longer would the problem of abiogenesis be one of *essence*, but rather one of *detail*. The major mystery at the heart of the OOL debate would be broadly resolved and the central issue would effectively be replaced by a variety of chemical questions that deal with the particular mechanisms by which those underlying principles could have been expressed. Issues such as identifying historic transitions, the definition of life, would become to some extent arbitrary and ruled by scientific conventions, rather than by matters of principle.

## 4. The role of autocatalysis during abiogenesis

In the context of the OOL debate, there is one single and central historic fact on which there is broad agreement—that life's emergence was initiated by some autocatalytic chemical system. The two competing narratives within the OOL's long-standing debate—‘replication first’ or ‘metabolism first’—though differing in key elements, both build on that autocatalytic character (see [1] and references therein). The ‘replication first’ school of thought stresses the role of oligomeric compounds, which express that autocatalytic capability through their ability to self-replicate, an idea that can be traced back almost a century to the work of Troland [19], while the ‘metabolism first’ school of thought emphasizes the emergence of cyclic networks, as articulated by Kauffman [20] in the 1980s and reminiscent of the metabolic cycles found in all extant life. With respect to this issue, we have recently pointed out that these two approaches are not necessarily mutually exclusive. It could well be that both oligomeric entities *and* cyclic networks were crucial elements during life's emergence, thereby offering a novel perspective on this long-standing question [1,7]. However, once it is accepted that autocatalysis is a central element in the process of abiogenesis, it follows that the study of autocatalytic systems in general may help uncover the principles that govern their chemical behaviour, regardless of their chemical detail. Indeed, as we will now describe, the generally accepted supposition that life's origins emerged from some prebiotic autocatalytic process can be shown to lead to broad insights into the chemistry–biology connection and to the surprising revelation that the processes of abiogenesis and biological evolution are directly related to one another. Once established, that connection will enable the underlying principles that governed the emergence of life on the Earth to be uncovered *without* undue reliance on speculative historic suppositions regarding the precise nature of those prebiotic systems.

## 5. A previously unrecognized stability kind: dynamic kinetic stability

The realization that the autocatalytic character of the replication reaction can lead to exponential growth and is unsustainable has been long appreciated, going back at least to Thomas Malthus's classic treatise ‘An essay on the principle of population’, published in 1798 [21]. But the chemical consequences of that long-recognized powerful kinetic character, although described by Lotka already a century ago [22], do not seem to have been adequately appreciated. Recently, one of us (A.P.) has described a new stability kind in nature, seemingly overlooked in modern scientific thought, which we have termed *dynamic kinetic stability* (*DKS*) [1,7,23,24]*.* That stability kind, applicable solely to persistent replicating systems, whether chemical or biological, derives directly from the powerful kinetic character and the inherent unsustainability of the replication process. However, for the replication reaction to be kinetically unsustainable, the reverse reaction, in which the replicating system reverts back to its component building blocks, must be very slow when compared with the forward reaction; the replication reaction must be effectively *irreversible.* That condition, in turn, means the system must be maintained in a *far-from-equilibrium* state [25], and that continuing requirement is satisfied through the replicating system being open and continually fed activated component building blocks. Note that the above description is consistent with Prigogine's non-equilibrium thermodynamic approach, which stipulates that self-organized behaviour is associated with irreversible processes within the nonlinear regime [26]. From the above, it follows that the DKS term would *not* be applicable to an *equilibrium* mixture of some oligomeric replicating entity together with its interconverting component building blocks.

Given the above discussion, it is apparent that the DKS concept is quite distinct from the conventional stability kind in nature, thermodynamic stability. A key feature of DKS is that it characterizes *populations of replicators*, rather than the individual replicators which make up those populations. Individual replicating entities are inherently *unstable*, as reflected in their continual turnover, whereas a population of replicators can be remarkably stable, as expressed by the persistence of some replicating populations. Certain life forms (e.g. cyanobacteria) express this stability kind in dramatic fashion, having been able to maintain a conserved function and a readily recognized morphology over billions of years. Indeed, within the world of replicators, there is theoretical and empirical evidence for a selection rule that in some respects parallels the second law of thermodynamics in that *less* stable replicating systems tend to become transformed into *more* stable ones [1,8]. This stability kind, which is applicable to all persistent replicating systems, whether chemical or biological, is then able to place biological systems within a more general physico-chemical framework, thereby enabling a physico-chemical merging of replicating *chemical* systems with *biological* ones. Studies in systems chemistry in recent years have provided empirical support for such a view by demonstrating that chemical and biological replicators show remarkably similar reactivity patterns, thereby reaffirming the existence of a common underlying framework linking chemistry to biology [1,7].

## 6. Extending Darwinian theory to inanimate chemical systems

**Scheme 1.**
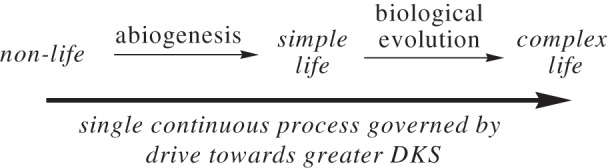
Unification of abiogenesis and biological evolution into a single continuous process governed by the drive toward greater DKS.

The recognition that a distinctly different stability kind, DKS, is applicable to both chemical and biological replicators, together with the fact that both replicator kinds express similar reaction characteristics, leads to the profound conclusion that the so-called chemical phase leading to simplest life and the biological phase appear to be one continuous physico-chemical process, as illustrated in scheme 1.

That revelation is valuable as it offers insights into abiogenesis from studies in biological evolution and, vice versa, it can provide new insights into the process of biological evolution from systems chemistry studies of simple replicating systems. A single continuous process necessarily means one set of governing principles, which in turn means that the two seemingly distinct processes of abiogenesis and evolution can be combined and addressed in concert. Significantly, that merging of chemistry and biology suggests that a general theory of evolution, expressed in physico-chemical terms rather than biological ones and applicable to both chemical and biological systems, may be formulated. Its essence may be expressed as follows: *All stable* (*persistent*) *replicating systems will tend to evolve over time towards systems of greater DKS.* As we have described in some detail in previous publications, there are both empirical and theoretical grounds for believing that oligomeric replicating systems which are *less* stable (less persistent) will tend to be transformed into *more* stable (more persistent) forms [1,7,8,24]. In fact that selection rule is just a particular application of the more general law of nature, almost axiomatic in character, that systems of *all* kinds tend from less stable to more stable. That law is inherent in the very definition of the term ‘stability’. So within the global selection rule in nature, normally articulated by the second law of thermodynamics, we can articulate a formulation specific to replicative systems, both chemical and biological—*from DKS less stable to DKS more stable*. A moment's thought then suggests that the Darwinian concept of ‘fitness maximization’ (i.e. less fit to more fit) is just a more specific expression of that general replicative rule as applied specifically to biological replicators. Whereas, in Darwinian terms, we say that living systems evolve to maximize fitness, the general theory is expressed in physico-chemical terms and stipulates that stable replicating systems, whether chemical *or* biological, tend to evolve so as to increase their stability, their DKS. Of course such a formulation implies that DKS is quantifiable. As we have previously discussed, quantification is possible, but only for *related* replicators competing for common resources, for example, a set of structurally related replicating molecules, or a set of genetically related bacterial life forms [1,7]. More generally, when assessing the DKS of replicating systems in a wider sense, one frequently must make do with qualitative or, at best, semi-quantitative measures.

Note that the general theory should not be considered as just one of changing terminology—‘DKS’ replacing ‘fitness’, ‘kinetic selection’ replacing ‘natural selection’. The physico-chemical description offers new insights as it allows the characterization of both the *driving force* and the *mechanisms* of evolution in more fundamental terms. The driving force is the drive of replicating systems towards greater stability, but the stability kind that is applicable in the replicative world. In fact that driving force can be thought of as a kind of second law analogue, though, as noted, the open character of replicating systems makes its quantification more difficult. And the mechanisms by which that drive is expressed can now be specified. These are *complexification* and *selection*, the former being largely overlooked in the traditional Darwinian view, while the latter is, of course, central to that view. A striking insight from this approach to abiogenesis follows directly: just as Darwinian theory broadly explained biological evolution, so an extended theory of evolution encompassing both chemical and biological replicators can be considered as broadly explaining abiogenesis. Thus, life on the Earth appears to have emerged through the spontaneous emergence of a simple (unidentified) replicating system, initially fragile, which complexified and evolved towards complex replicating systems exhibiting greater DKS. In fact, we would claim that in the very broadest of terms, the physico-chemical basis of abiogenesis can be considered explained.

But does that simplistic explanation for abiogenesis imply that the OOL problem can be considered resolved? Far from it. Let us now consider why.

## 7. What is still to be learned?

While Darwin's revolutionary theory changed our understanding of how biological systems relate to one another through the simple concept of natural selection, the Darwinian view has undergone considerable refinement and elaboration since its proposal over 150 years ago. First the genomic revolution, which provided Darwin's ideas with a molecular basis through the first decades of the twentieth century, transformed the subject and led to the neo-Darwinian synthesis, an amalgamation of classic Darwinism with population genetics and then with molecular genetics. But in more recent years, there is a growing realization that a molecular approach to understanding evolutionary dynamics is insufficient, that evolutionary biology's more fundamental challenge is to address the unresolved problem of complexity. How did biological complexity come about, and how can that complexity and its dynamic nature be understood? Our point is that Darwin's monumental thesis, with natural selection at its core, was just the beginning of a long process of refinement and elaboration, which has continued unabated to the present day.

Precisely the same process will need to operate with respect to the OOL problem. The DKS concept, simple in essence, does outline in the broadest terms the physico-chemical basis for abiogenesis. But that broad outline needs to be elaborated on through experimental investigation, so that the detailed mechanisms by which the DKS of simple chemical replicating systems could increase would be clarified. Already at this early stage, central elements of those mechanisms are becoming evident. Thus, there are preliminary indications that the process of abiogenesis was one of DKS enhancement through complexification [1,7]. More complex replicating systems, presenting a diversity of features and functions, appear to be able to replicate more effectively than simpler ones, and so are likely to be more stable in DKS terms (though this should not be interpreted to mean that *any* form of complexification will necessarily lead to enhanced DKS). The pertinent question is then: how does that process of complexification manifest itself? And this is where systems chemistry enters the scene [9–11]. By studying the dynamics of simple replicating molecular systems and the networks they establish, studies in system chemistry are beginning to offer insights into that process of replicative complexification. Following on from earlier work by Sievers & von Kiedrowski [27] and Lee *et al*. [28], more recent studies on RNA replicating systems by Lincoln & Joyce [29] and most recently by Vaidya *et al*. [30] suggest that network formation is crucial. Thus, Lincoln & Joyce [28] observed that a molecular network based on *two* cross-catalysing RNAs replicated rapidly and could be sustained indefinitely. By contrast, the most effective *single* molecule RNA replicator replicated slowly and was not sustainable. But in a more recent landmark experiment, Vaidya *et al*. [30] demonstrated that a cooperative cycle made up of three self-replicating RNAs could out-compete those same RNAs acting as individual replicators. The conclusion seems clear: molecular networks are more effective in establishing self-sustainable autocatalytic systems than single molecule replicators, just as was postulated by Eigen & Schuster [25] some 40 years ago.

Many key questions remain unanswered, however. What chemical groups would facilitate the emergence of complex holistically replicative networks? Are nucleic acids essential for the establishment of such networks, or could other chemical groups also express this capability? Is template binding the main mechanism by which molecular autocatalysis can take place, or can holistically autocatalytic sets be established through cycle closure without a reliance on template binding? How would the emergence of individual self-replicating entities *within* a larger holistically replicative network contribute to the stability of the network as a whole? How do kinetic and thermodynamic factors inter-relate in facilitating the maintenance of dynamically stable, but thermodynamically unstable, replicating systems [12,13]? As these questions suggest, our understanding of central issues remains rudimentary, and the road to discovery will probably be long and arduous. However, the key point of this essay has been to note that just as Darwin's simple concept of natural selection was able to provide a basis for an ongoing research programme in evolution, one that has been central to biological research for over 150 years, so the DKS concept may be able to offer a basis for ongoing studies in systems chemistry, one that may offer new insights into the rules governing evolutionary dynamics in simple replicating systems and, subsequently, for replicating systems of all kinds. Such a research programme, we believe, promises to further clarify the underlying relationship linking chemical and biological replicators.

In conclusion, it seems probably that we will never know the precise *historic* path by which life on the Earth emerged, but, very much in the Darwinian tradition, it seems we can now specify the essence of the *ahistoric* principles by which that process came about. Just as Darwin, in the very simplest of terms, pointed out how natural selection enabled simple life to evolve into complex life, so the recently proposed general theory of evolution [1,7] points out in simplest terms how simple, but fragile, replicating systems could have complexified into the intricate chemical systems of life. But, as discussed earlier, a detailed understanding of that process will have to wait until ongoing studies in systems chemistry reveal both the classes of chemical materials and the kinds of chemical pathways that simple replicating systems are able to follow in their drive towards greater complexity and replicative stability.
